# The epigenome of male germ cells and the programming of phenotypes in cattle

**DOI:** 10.1093/af/vfab062

**Published:** 2021-12-17

**Authors:** Hélène Kiefer, Eli Sellem, Amélie Bonnet-Garnier, Maëlle Pannetier, Valentin Costes, Laurent Schibler, Hélène Jammes

**Affiliations:** 1 Université Paris-Saclay, UVSQ, INRAE, BREED, 78350, Jouy-en-Josas, France; 2 Ecole Nationale Vétérinaire d’Alfort, BREED, 94700, Maisons-Alfort, France; 3 R&D Department, ALLICE, 149 rue de Bercy, 75012, Paris, France

**Keywords:** cattle, DNA methylation, embryo development, epigenetics, male germ cells, small noncoding RNAs

**Implications**

- Bull semen is a commercial product widely used for artificial insemination.
- During the differentiation of male germ cells into spermatozoa, there are several windows of epigenome sensitivity to environmental factors.
- The epigenome of bull sperm exhibits both conserved features and interindividual variations, some of which are associated with fertility.
- The paternal epigenome contributes to embryo development and to programming the phenotype of offspring.

## Introduction 

DNA methylation plays an important role in the structure and stability of the genome when associated with heterochromatin and repetitive elements such as transposable elements (**TE**s; mobile elements in the genome of viral origin that have accumulated during evolution) and satellites (sequences located near or at the centromeric regions of chromosomes). DNA methylation also regulates transcription and is dynamic as a function of cell type, developmental stage, the animal’s physiology, or the environment. Finally, DNA methylation is involved in the genomic imprinting of genes expressed in a mono-allelic manner depending on the parental origin of the allele, a phenomenon that is essential for harmonious growth of the fetus. DNA methylation is catalyzed by DNA methyltransferases (**DNMT**s) that use S-adenosylmethionine as a methyl donor (produced from dietary folic acid). DNMT3A and DNMT3B enzymes are involved in de novo DNA methylation, while DNMT1, which recognizes the hemi-methylated cytosine-phosphate-guanine (**CpG**) sites resulting from DNA replication, ensures the maintenance and propagation of methylation patterns through cell division. The erasure of DNA methylation can result not only from a lack of DNMT1 activity but also from the conversion of 5-methylcytosines (**5meC**s) by ten-eleven translocation (**TET**) enzymes into oxidized derivatives such as 5-hydroxymethylcytosine (**5hmC**), which are then diluted during replication or replaced by unmethylated cytosines by the DNA repair machinery. Genomic DNA is wrapped around octamers of histones to form the nucleosome. Histones contain N-terminal tails targeted by different types of modifications, such as acetylation and methylation. These modifications affect different amino acids, producing dozens of posttranslational variants with different functional roles. The addition or removal of these modifications is highly flexible processes that directly affect the accessibility of genomic DNA to the transcription machinery and hence the activation or repression of gene expression. The combinatorial nature of the different histone marks, together with DNA methylation and the presence of certain transcription factors or RNA polymerase, define specific chromatin states associated with specific transcriptional states. These chromatin states are transmitted to daughter cells, thus ensuring the continuity of cell identity through mitosis. Finally, small noncoding RNAs (**sncRNA**s) play an important role in posttranscriptional regulations, and their expression is tightly regulated in a cell type-specific manner.

Mature spermatozoa are transcriptionally inactive and represent the ultimate form of male germ cell (**GC**) differentiation. Their fate is to survive outside the organism and contribute to a new individual after fertilization of an oocyte. In support of these functions, the epigenome of spermatozoa is unique (Carrell, 2012). Depending on the species, 85% to 99% of the histones are replaced by protamines, arginine-rich proteins that form toroid-shaped structures with DNA. This replacement enables a higher level of chromatin compaction, which contributes to reducing nuclear volume and helps to protect the paternal genetic heritage against oxidation during migration through the epididymis and female genital tract (Champroux et al., 2016). Furthermore, in addition to microRNAs (**miRNA**s) and small interfering RNAs (**siRNA**s) that are also found abundantly in somatic cells, the germline is enriched in P-element induced wimpy testis (**PIWI**)-interacting RNAs (**piRNA**s). 

**Figure 1.**
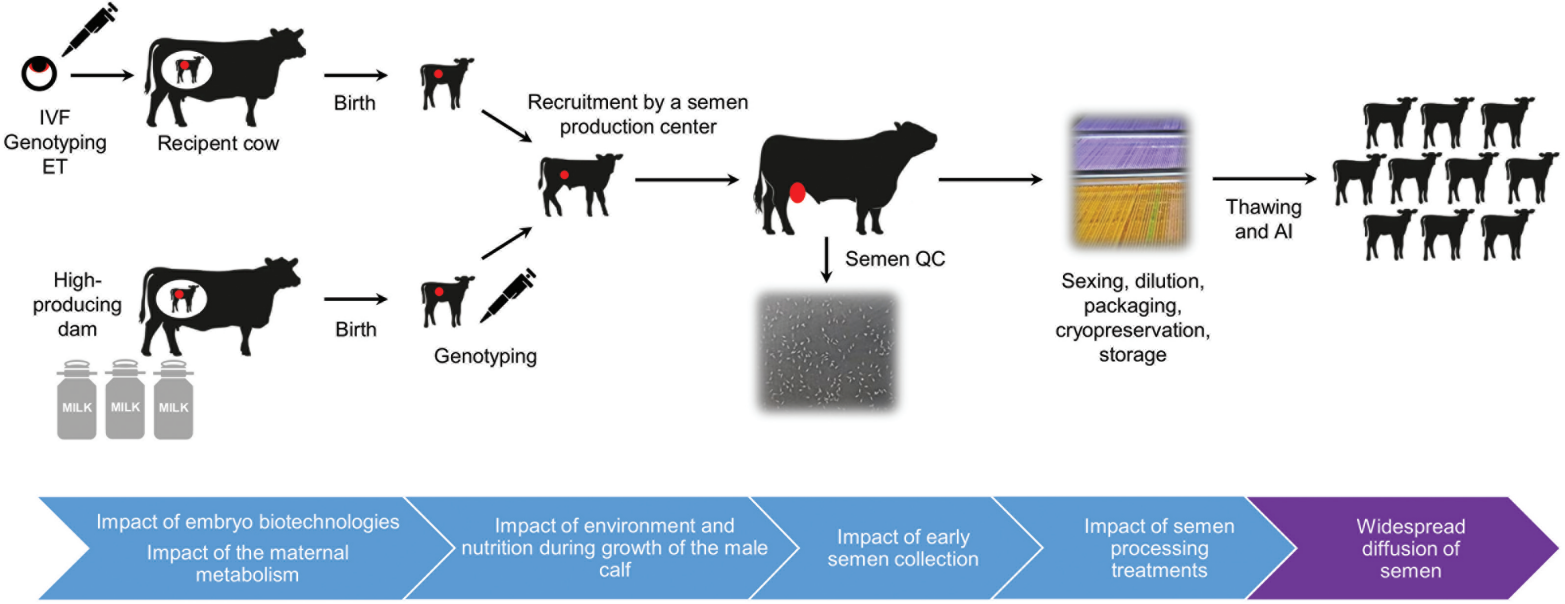
Breeding, selection, and semen processing practices in the cattle AI industry. The different steps that may impact the sperm epigenome of bulls are highlighted. The developing germline is shown in red. ET, embryo transfer; IVF, in vitro fertilization; QC, quality control.

The sperm-specific epigenome is acquired during the differentiation of male GCs into mature spermatozoa, a process that starts at the embryonic stage and is only achieved after puberty has been reached and during each cycle of spermatogenesis (Carrell, 2012; Champroux et al., 2016). In cattle and especially dairy breeds, where bull semen is widely used for artificial insemination (**AI**), several selection and breeding practices may interfere with proper establishment of the sperm epigenome (Figure 1). The selection of AI bulls relies on their genetic merit, and they are usually obtained from the breeding of high breeding value sires and high-producing dairy cows. These cows are more likely to experience a negative energy balance in the event of concurrent lactation and gestation, which may lead to an unfavorable in utero environment for the developing fetus (Wu and Sirard, 2020). Otherwise, practices to reduce the generation interval and accelerate genetic gain, such as hormonal treatments of the mothers, embryo technologies, or the hastened growth and puberty of male calves, may have a long-term impact on the sperm epigenome (Rivera, 2019). Finally, bull semen is extensively processed before its use for AI, which, according to data obtained in other species, may affect the chromatin structure (Aurich et al., 2016).

Because bull semen has a widespread diffusion potential, with dozens of offspring potentially being generated per batch, it is important to understand the impact of these practices on the epigenetic landscape of spermatozoa and the degree to which variations in the epigenome might affect fertility and the phenotype of offspring. The goal of this short review is, therefore, to provide an overview of recent knowledge regarding the epigenome of male GCs and its potential role in the programming of phenotypes, with particular emphasis on cattle and in light of the knowledge accumulated in other species.

## Epigenetic Reprogramming of Male GCs and Windows of Sensitivity to Environmental Factors

Establishment of the male germline requires three successive stages: 1) specification of primordial GCs (**PGC**s) from the embryonic epiblast, 2) migration and colonization of the genital ridges that will form the testes, and 3) differentiation into male GCs (pro-spermatogonia or gonocytes), which stop proliferating and enter quiescence. After birth, the male GCs resume mitosis and progressively migrate from the center to the basement of seminiferous cords. In parallel with these processes, the pool of spermatogonial stem cells (**SSC**s), from which spermatogenesis is sustained over a lifetime, is gradually established from male GCs. The whole process of spermatogenesis only becomes effective after puberty and comprises a mitotic phase (spermatogonia), a meiotic phase (spermatocytes), and spermiogenesis (spermatids). During this last step, dramatic morphological changes occur that convert round and transcriptionally active spermatids into spermatozoa harboring a flagellum, a head, a tightly compacted nucleus, an acrosome, and almost no cytoplasm. Spermatozoa are then released into the lumen of the seminiferous tubules and transit through epididyma where they acquire motility and complete their maturation until fertilization (Wrobel, 2000; Staub and Johnson, 2018).

**Figure 2.**
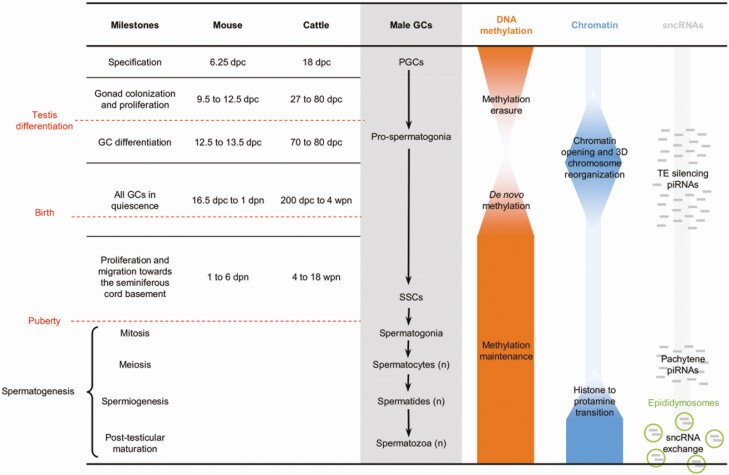
Epigenetic reprogramming during the differentiation of male GCs in mice. The timing of the different milestones in cattle has been established using unpublished data from our lab and from the study of Wrobel (2000). The timing of epigenetic reprogramming in bovine fetuses is unknown. dpc, day post coitum; dpn, day postnatal; wpn, week postnatal.

These differentiation and maturation processes are based on a specific transcriptional program orchestrated by extensive epigenetic reprogramming (Figure 2). Most knowledge concerning the reprogramming of DNA methylation has been acquired in mice and, to a lesser extent, in humans. The DNA methylation pattern that characterizes the epiblast is thus erased throughout the genome when PGCs colonize the gonad. DNA methylation erasure involves mechanisms that are both passive (through cell division and the absence of maintenance activity) and active (through the generation of 5hmC). This erasure is not total: some genomic regions retain methylation to a degree that differs as a function of species (Tang et al., 2016). In porcine male GCs, persistent DNA methylation is observed in some TEs and in overlapping genes (Gómez-Redondo et al., 2021), suggesting that the lack of DNA methylation erasure in these genes is a safeguard against the mobilization of TEs. In parallel with DNA demethylation, levels of repressive histone marks rise, which prevents the initiation of massive transcriptional activity following genome hypomethylation (Tang et al., 2016). The loss of 5meC and then of repressive histone marks at specific loci, as well as the gain in 5hmC, are all essential for the expression of germline differentiation genes and thus establishment of the germline (Hill et al., 2018).

The DNA re-methylation of GCs uses the de novo methylation enzymes DNMT3A/3B and DNMT3L; the latter is a germline-specific cofactor that is devoid of methyltransferase activity and guides DNMT3A/3B to the sequences to be methylated. In mice, the bulk of de novo DNA methylation occurs during the period of male GC quiescence. In regions associated with euchromatin, the broad deposit of H3K36me2 histone mark, which is recognized and bound by DNMT3A, is necessary for the first wave of de novo DNA methylation (Shirane et al., 2020). In heterochromatin, de novo methylation is delayed and appears to rely on a broad reorganization of chromatin occurring later during mouse development (Yamanaka et al., 2019). PIWI-interacting RNAs contribute to de novo DNA methylation through their role in silencing TEs which try to invade the genome of the germline to be propagated at the next generation. To achieve this silencing, piRNAs displaying partial sequence homology with TEs guide the recruitment of de novo DNA methylation and chromatin remodeling machineries toward nascent TE transcripts (Wang and Lin, 2021). Although most 5meC in male GCs is acquired during life in utero, it appears that modifications still occur after birth (Oakes et al., 2007). Data obtained in mice suggest that the rate and distribution of 5meC in male GCs then stabilize before meiosis, because no important changes can be observed between spermatocytes and spermatozoa (Oakes et al., 2007; Hammoud et al., 2014).

As largely supported by data in mice, the genome-wide erasure and re-apposition of DNA methylation are an important window of epigenetic plasticity that can be altered by deleterious environmental conditions. The living conditions of the mother may affect the reprogramming of male GCs and the sperm methylome in adulthood, with possible physiological effects on reproductive outcomes (Lambrot et al., 2013) and on the metabolism of the next generation (Martínez et al., 2014). In sheep, nutritional stress during pregnancy alters the DNA methylation landscape and the functional parameters of spermatozoa (Toschi et al., 2020). The dynamics of de novo DNA methylation are not yet understood in male cattle; however, the sperm methylome retains a memory of the nutrition offered during the first months of life (Perrier et al., 2020), suggesting that DNA methylation after birth is still sensitive to environmental factors. Likewise, environmental control during gestation (and particularly the diet of highly producing dams) may prove crucial to ensuring the proper differentiation of male GCs, optimal fertility traits, and an adequate sperm methylome throughout adulthood.

In contrast with the overall stability of DNA methylation during adulthood, chromatin and sncRNA contents are dynamically remodeled during spermatogenesis and beyond. Micro RNAs play an important role in spermatogenesis in mice; they are involved in regulating the differentiation vs. proliferation balance in SSCs (Huang et al., 2017) as well as meiosis and the histone–protamine transition (Liu et al., 2013). Functions in regulation of the stability and translation of mRNAs during mouse spermatogenesis have also been reported for piRNAs, as well as a role in chromosome segregation during meiosis through the regulation of RNAs produced from satellite repeats (Wang and Lin, 2021). Since post-spermiogenesis spermatozoa are transcriptionally inactive, their sncRNAs content was long thought to be stable and exclusively inherited from spermatogenesis. However, it has recently been demonstrated in several species (Chu et al., 2019; Nixon et al., 2019; Sellem et al., 2021) that the sncRNA profile of sperm undergoes important modifications in contact with extracellular vesicles trafficked from epithelial cells in the epididymis (epididymosomes). The piRNA content thus falls markedly during epididymal transit and is replaced by other sncRNA families. In bulls, miRNAs account for 1% of the testicular sperm sncRNA content and then rise to reach 30% in epididymis cauda. The proportion of transfer RNAs- (tRFs) or ribosomal RNAs- (rRFs) derived fragments also increases rapidly as the spermatozoa reach the epididymis (Sellem et al., 2021). The transit of spermatozoa through the epididymis, therefore, represents an important window of epigenetic plasticity, which could be mediated by changes to the sncRNA content of epididymosomes depending on environmental or physiological factors. In line with this view, modifications to the sncRNA profile of sperm have been reported in response to diet in rodents (Grandjean et al., 2015; de Castro Barbosa et al., 2016).

## The Epigenome of Bull Sperm and Its Relationships with Fertility

Mature bovine spermatozoa have a particularly low global level of 5meC compared with bovine somatic cells and also to spermatozoa from goats, rams, humans, stallions, boars, and mice. This low 5meC level has been observed in all cattle breeds studied to date and does not seem to be affected by the semen freezing process (Perrier et al., 2018). To determine the undermethylated sequences, the sperm methylome was compared with that of bovine somatic cells using pan-genomic approaches (reduced representation bisulfite sequencing and the immunoprecipitation of methylated DNA followed by hybridization on a microarray). Numerous differentially methylated positions were found, 81% of which were specifically undermethylated in spermatozoa. These undermethylated sites are enriched with spermatogenesis genes and satellite repeats. Overrepresentation of these repeats in the bovine genome, as well as their low methylation, may explain why bovine spermatozoa have lower global 5meC levels than spermatozoa from other mammalian species. Using a different whole-genome approach (whole-genome bisulfite sequencing), another team reported that the DNA methylome of bull sperm contains specific undermethylated domains enriched for satellites and evolutionary young TEs that may escape piRNA-mediated silencing (Zhou et al., 2018). The lower methylation of satellites in sperm compared with somatic cells has been reported in other species (Qu et al., 2018); however, the difference seems to be particularly marked in the bovine species.

The silencing of pericentromeric satellites by the formation of constitutive heterochromatin is essential to maintaining genome stability and preventing both recombination and inappropriate chromosome segregation. In somatic cells, pericentromeric heterochromatin formation is primarily achieved through DNA methylation and the recruitment of heterochromatin protein HP1 to the H3K9me3 histone mark. Some components of the somatic constitutive heterochromatin appear to be maintained in mouse sperm, since satellites escape genome-wide histone–protamine exchange and remain associated with nucleosomes bearing H3K9me3 (Yamaguchi et al., 2018). In addition, histones are detected in distal intergenic regions and CpG-rich promoters and those of developmentally important genes. In bull sperm, two studies have reported partially concordant results regarding the genome-wide location of nucleosomes; interestingly, both agreed to confirm the retention of histones at satellites (Samans et al., 2014; Sillaste et al., 2017).

An exhaustive analysis of the expression profiles of sperm sncRNAs in a cohort of 40 bulls from six breeds was recently carried out (Sellem et al., 2020). Several sncRNA families were detected, including miRNAs (20%), piRNA (26%), rRFs (25%), and tRFs (14%). Interestingly, tRFs associated with glycine or glutamine and derived from the 5′ half of tRNAs were highly represented among all tRNAs. Whatever the sncRNA family, few sequences were predominantly expressed. For instance, the 20 most expressed miRNA sequences accounted for 75% of total miRNA expression, suggesting their functional importance. Numerous isomiRs (sequence variants of canonical miRNAs) were also identified, thus increasing the diversity and complexity of the bull sperm sncRNA repertoire. These variations were not related to the presence of known genetic polymorphisms, suggesting that they could rely on specific RNA edition mechanisms such as the trimming or adding of one or several nucleotides at sequence extremities. Such edition mechanisms have been described in humans, where several nucleotidyl transferases (especially uridyltransferases and adenyltransferases) are involved in the biogenesis of isomiRs (Neilsen et al., 2012). Among all the sequences identified as miRNAs, only 26% have been described and recorded in databases, suggesting that bull sperm contains many novel and putative miRNAs. Such diversity in the sncRNA content of bull sperm has thus been reported for the first time and was probably determined, thanks to an optimized RNA extraction method and important sequencing depth (Sellem et al., 2020).

Bull semen is a commercial product widely used for AI. Because unsuccessful AI can result in economic losses, extended calving intervals, increased culling rates, and lower rates of genetic gain, several studies (listed below) have investigated the association between interindividual variations of the semen epigenome and fertility traits of bulls. Some studies have highlighted differences in DNA methylation patterns between groups of bulls with different fertility scores (Kropp et al., 2017; Gross et al., 2020; Narud et al., 2021; Takeda et al., 2021). Overall, the results reported were not concordant in terms of the genes or genomic regions targeted by differential methylation and the magnitude of DNA methylation changes. These inconsistencies may be related to both technical issues (e.g., because of the different approaches used to generate DNA methylation data and parameters used to detect differential methylation) and biological issues (different scores used to assess bull fertility, different breeds, high interindividual variability, and small numbers of samples involved in each study). Likewise, several studies have focused on the association between interindividual variations in the sperm sncRNA content and semen quality or bull fertility, highlighting several miRNAs (Capra et al., 2017; Alves et al., 2020; Keles et al., 2021). In addition, other sncRNAs such as tRF-Gly or tRF-Glu may represent another source of fertility biomarkers, as suggested by their differential expression according to in vitro fertilization outcomes in humans (Hua et al., 2019). Due to the high compaction level of sperm chromatin, studies on the genomic location of posttranslational modifications of histones are technically challenging; however, the histone retention degree and associated modifications have been reported to vary as a function of bull fertility using flow cytometry (Ugur et al., 2019).

Because of its multifactorial nature, understanding and predicting fertility are challenging. Furthermore, in the AI industry, routine semen quality control tests are carried out (Figure 1), allowing the identification of most bulls with severe infertility and observable effects on semen functional parameters. Compared with humans, the difference between fertile and subfertile bulls is subtle, hampering the accurate prediction of fertility. For these reasons, most of the studies mentioned above should be regarded as prospective. Larger cohorts of bulls that are well characterized in terms of their genotypes and fertility would be necessary to reduce interindividual variability and to develop models. Integrating various signals (DNA methylation, sncRNAs, and genotypes) rather than considering only one source of information may also provide additional insights into the architecture of fertility. Because spermatozoa are transcriptionally silent, another avenue could arise from functional experiments on the embryo, such as monitoring the effects of the overexpression or suppression of particular miRNAs on the kinetics and quality of embryonic development. This research could have potential applications in human medicine, as AI bulls usually have hundreds of AI records, which considerably alleviates confounding effects and enables the very precise assessment of male fertility.

## Contribution of the Paternal Epigenome to Embryonic Development

**Figure 3.**
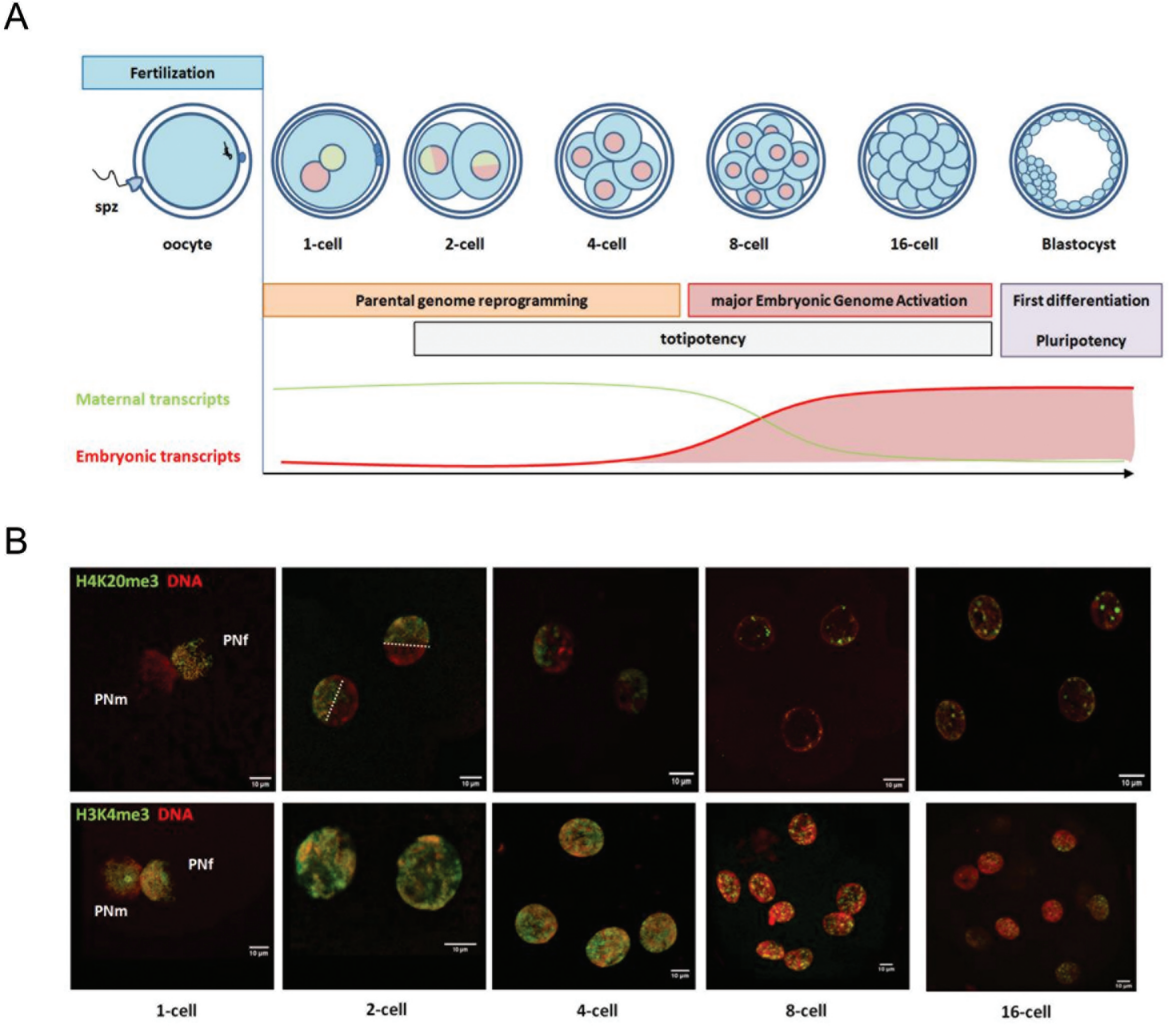
Reprogramming of the parental epigenome during embryonic development in cattle. (A) Upper panel: schematic representation of early embryonic development from fertilization to the first differentiation. Lower panel: dynamics of maternal (green) and embryonic (red) transcripts. (B) Distribution of H4K20me3 (upper panel) and H3K4me3 (lower panel) in the nuclei of bovine embryos from 1-cell to 16-cell stages (unpublished data from our lab). Scale bar: 10 µm. PNf, female pronucleus; PNm, male pronucleus; spz, spermatozoa.

Evidence demonstrating that the epigenetic information carried by gametes is crucial for development is provided by the poor developmental outcomes of clones (Heyman, 2005). Cloned zygotes are obtained by the transfer of a somatic cell into an enucleated oocyte; they are, therefore, diploid but lack paternal and maternal epigenomes as well as the whole sncRNA content of sperm. The somatic epigenome represents the main barrier to the efficiency of cloning, while the oocyte and sperm epigenomes are extensively reprogrammed after fertilization to allow development of the embryo (Figure 3A).

This reprogramming is characterized by a series of epigenetic modifications that start just after fertilization (Ross and Sampaio, 2018), particularly in the paternal genome. The protamines present on paternal DNA are exchanged with maternal histones, which are rapidly methylated in position H3K4 (activating mark; see Figure 3B, lower panel). By contrast, the maternal chromatin contains numerous repressive histone modifications such as H3K9me3, H3K27me3, and H4K20me3 (Figure 3B, upper panel). This asymmetry between parental genomes eventually fades as embryonic development progresses (Bošković et al., 2012). Overall 5meC levels fall sharply in embryos during preimplantation development (Lepikhov et al., 2008; Dobbs et al., 2013; Bakhtari and Ross, 2014). Genes subject to genomic imprinting are not affected by this wave of DNA demethylation (Jiang et al., 2018; Duan et al., 2019). DNA methylation erasure is more rapid and important in the bovine paternal genome (which is initially more methylated than the maternal genome) and requires the expression of TET enzymes (Bakhtari and Ross, 2014). At the level of the genomic sequence, this overall 5meC decrease is associated with three successive waves of DNA demethylation and de novo methylation. The three steps of DNA methylation erasure coincide with the principal stages of early development and the expression of specific genes, including those encoding de novo DNMTs, which may explain why they are followed by de novo DNA methylation (Jiang et al., 2018).

The epigenetic changes affecting chromatin and DNA methylation participate in triggering embryonic genome activation (**EGA**). Indeed, initially, the genome of the newly fertilized embryo is transcriptionally inactive. Embryo development then depends strictly on the stock of RNA and proteins accumulated in the oocyte (Figure 3A). EGA occurs at the 8-cell stage in cattle and relies on a unique chromatin organization. The overall levels of repressive histone marks reach a minimum level at EGA and recover to the blastocyst stage, as the first cell differentiation occurs. Chromatin accessibility in bovine embryos is also maximal at the time of EGA, and several waves of transcription factor binding sites become accessible from the 2-cell to the morula stages, according to a dynamics that is closer to humans than to mice (Halstead et al., 2020). Likewise, the minimal level of DNA methylation (15%) is coincident with EGA (Duan et al., 2019).

Up to EGA, protein synthesis from the maternal mRNA stock can be regulated by sncRNAs originating from both the oocyte and spermatozoa. These gametic mRNAs and sncRNAs are then progressively diluted as the embryo starts transcribing its own material (Alves et al., 2020). Although the window during which the sperm-borne sncRNAs might exert a regulatory role in the embryo is narrow, they appear to be essential to the normal development of mouse embryos. This was recently illustrated by the developmental arrests and altered transcriptome exhibited by mouse embryos that had been produced using spermatozoa collected in the caput epididymis, which were, therefore, immature regarding their sncRNA content (Conine et al., 2018). Interestingly, the incubation of these immature spermatozoa with cauda epididymosomes restored normal embryonic development, thus demonstrating that essential factors, which may include sncRNAs, are embedded in these epididymosomes.

## Potential Mechanisms for the Programming of Phenotypes via the Paternal Route

The sperm epigenetic features transmitted to the embryo are postulated to mediate the intergenerational transmission of nongenetic information that may impact the long-term phenotype of offspring in response to environmental changes affecting the father (Champroux et al., 2018; Donkin and Barrès, 2018). Overall, studies on postfertilization reprogramming in cattle (Duan et al., 2019) and other species have suggested a limited inheritance of methylated features in the paternal genome. However, individual loci, such as imprinted loci, specific subfamilies of TEs, as well as a few genes, are specifically targeted by the DNA methylation maintenance machinery and are faithfully maintained throughout postfertilization reprogramming in mice (Seah and Messerschmidt, 2018). It has recently been demonstrated in mice that unmethylated CpGs bound by transcription factors expressed in male GCs or in the embryo are protected from de novo DNA methylation, while the absence of transcription factor binding at CpGs already methylated before reprogramming would allow the faithful re-apposition of DNA methylation (Kremsky and Corces, 2020). The authors of this study proposed that any change affecting the expression or binding of these transcription factors during de novo DNA methylation phases may, therefore, stably switch the methylation status of neighboring CpGs, thus offering a novel hypothesis for the mechanistic basis of epigenetic inheritance.

Small noncoding RNAs may also play a role in intergenerational inheritance, as exemplified by studies on the impact of diet on the F0 sncRNA content in rodent sperm. The F1 generation produced with this epigenetically altered sperm is affected by metabolic disorders and displays a modified sperm sncRNA content. Few sncRNAs, such as let-7c, are dysregulated in both F0 and F1 generations. Interestingly, among the let-7c targets, several genes are involved in glucose metabolism and could contribute to the phenotype observed (de Castro Barbosa et al., 2016). Pups developed from zygotes microinjected with the miRNA miR-19b exhibit metabolic alterations similar to those of pups sired by males fed a high-fat diet, suggesting that this particular miRNA instructs the paternal effect induced by diet (Grandjean et al., 2015). Likewise, protein restriction increases the amounts of tRF-Gly in sperm, which in turn modulates transcription in the embryo (Sharma et al., 2016).

Although most histones are removed from the sperm chromatin, those that are retained bear epigenetic marks that could be transmitted to the embryo and mediate intergenerational epigenetic effects (Champroux et al., 2018). In support of this hypothesis, a recent study demonstrated that the distribution of H3K4me3 was altered in the sperm of mice fed a folate-deficient diet from weaning to adulthood. Some of these alterations were also found in the embryos produced using this sperm, which may underlie changes to the post-EGA transcriptome and ultimately lead to developmental defects (Lismer et al., 2021).

**Figure 4.**
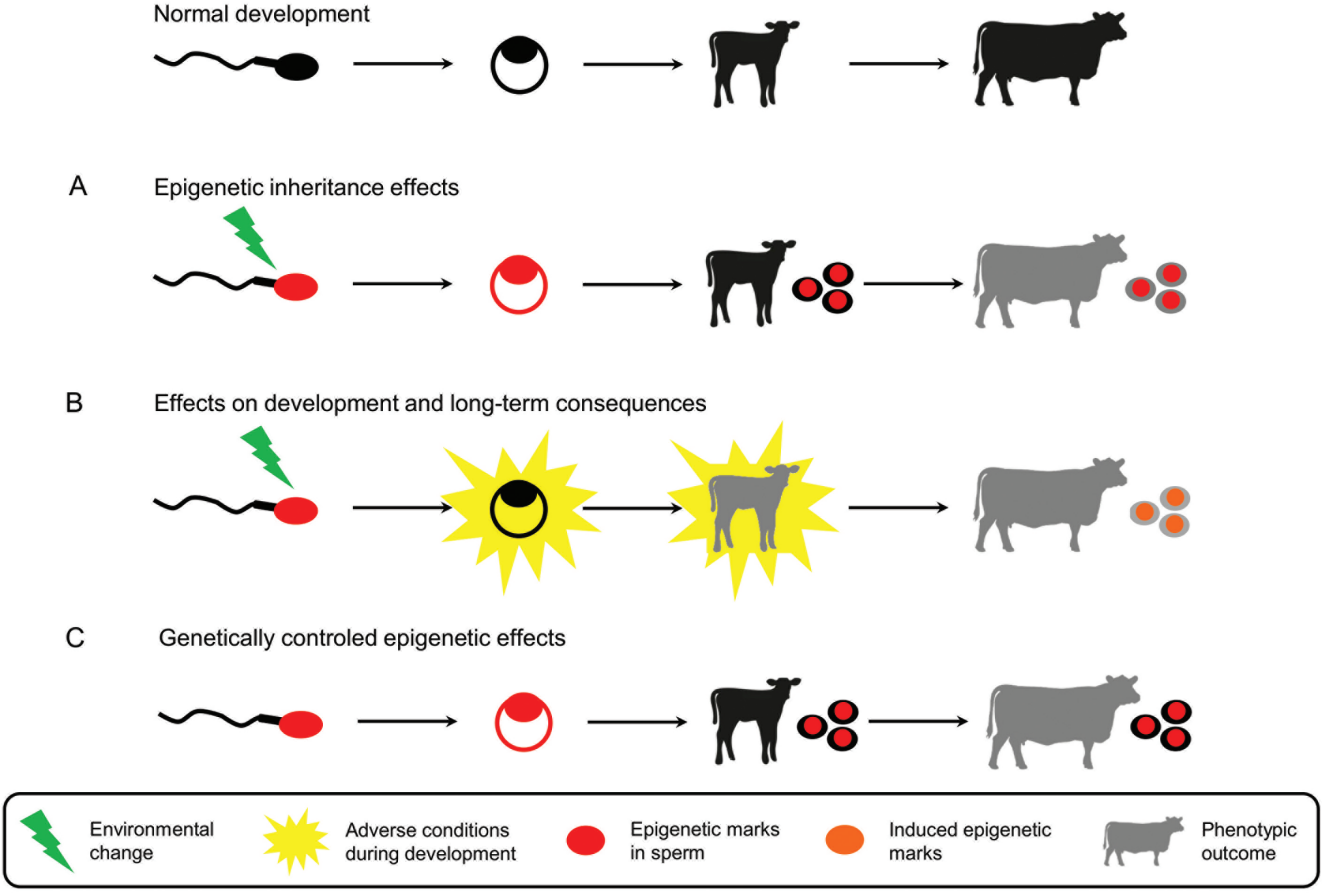
Simplified model for paternal effects on the offspring phenotype in cattle. Environmentally induced epigenetic changes in sperm can either be inherited by the offspring and drive phenotypic changes in the short or longer term (A) or interfere with normal development without transmission (B). The epigenetic marks thus induced and observable in the offspring are then the consequence rather than the cause of adverse conditions during development. (C) The epigenetic marks in sperm can also be transmitted to offspring through genetic mechanisms, leading to phenotypic outcomes independently of environmental changes. These genetically controlled epigenetic effects can easily be confounded with epigenetic inheritance.

Such epigenetic inheritance phenomena via the paternal route have so far not been reported convincingly in cattle but would be of considerable interest in the context of animal selection. Consistent with the major epigenetic reprogramming steps that occur during GC differentiation and after fertilization, modeling approaches have suggested that the overall magnitude of epigenetic inheritance is weak in cattle (Varona et al., 2015). Beyond epigenetic inheritance (Figure 4A), other molecular mechanisms involving the sperm epigenome may also modulate the offspring phenotype. For instance, aberrant epigenetic patterns or sncRNA contents in sperm may interfere with postfertilization reprogramming and alter the timing of the activation of developmentally regulated genes. The resulting embryo may carry subtle molecular, morphological, or metabolic defects that could drive long-term effects on the phenotype, in line with the theory regarding the developmental origin of health and diseases (**DOHaD**, Figure 4B). For instance, the exposure of male GCs to heat stress during spermatogenesis leads to chromatin condensation defects in bull spermatozoa and interferes with the reprogramming of DNA methylation in the paternal pronucleus after fertilization (Rahman et al., 2014). Another example is provided by the epigenetic alterations that have been observed in semen collected at a peripubertal age. Embryos produced using such peripubertal semen display subtle modifications to the DNA methylome and transcriptome that particularly affect the genes involved in metabolic functions and protein synthesis (Wu and Sirard, 2020). It is noteworthy that only morphologically normal embryos, which would likely have implanted and given birth to progeny, were considered during this study. Although later developmental stages were not investigated in these examples, it is possible that epigenetic changes induced by altered preimplantation development might be detected in the offspring. Lastly, some epigenetic features in sperm may be inherited by offspring because they are controlled by genetic mechanisms. Like other genetically controlled phenotypes, these epigenetic features are expected to be heritable. Because selection in cattle is reliant on the association between sire genotypes and daughter performances, it appears particularly challenging to disentangle epigenetic inheritance from genetically controlled epigenetic effects. A clearer understanding of the proportion of the epigenome under genetic control is, therefore, essential if we are to produce an initial estimate of intergenerational epigenetic inheritance in cattle and its impact on phenotypes.

## Conclusions

Numerous studies conducted in humans or model species have established the role of in utero conditions in the long-term programming of phenotypes, a phenomenon known as DOHaD. The concept of Paternal Origins of Health and Diseases has emerged more recently (Soubry, 2018), underscoring the importance of the paternal epigenome to offspring phenotype. In cattle, several selection and breeding practices may interfere with proper establishment of the sperm epigenome of bulls used for AI, leading to epigenetic alterations that can potentially disseminate to many herds and have long-term consequences. Windows of epigenome sensitivity to environmental factors exist, during which particular attention should be paid to the bull and its environment in order to maximize the epigenetic potential of sperm. Optimal in utero conditions should first be put in place by monitoring the nutrition, health, and welfare of the dam in order to ensure the proper epigenetic reprogramming and development of fetal GCs. Optimal conditions during the postnatal period, with particular focus on the transition phases, growth and puberty, as well as during the semen production period, should contribute to early and efficient spermatogenesis, the stable production of high-quality semen, and the overall fertility of the bull. The early culling of AI bulls with poor semen functional parameters is a common practice in the breeding industry, resulting in the elimination of males with severe infertility and thus facilitating the distribution of semen devoid of major epigenetic defects to different herds. On the other hand, the development of embryo biotechnologies such as intra-cytoplasmic sperm injection can to some extent compensate for spermatogenesis defects. As a consequence, bulls with exceptional genetic merit but poor semen quality can now be used in breeding schemes to generate marketed AI bulls whose semen will in turn be distributed extensively to herds. The intergenerational impact of embryo biotechnologies, combined with poor semen quality involving probable epigenetic defects, is still a matter of debate in humans and should also be considered in livestock. The degree to which nongenetic factors carried by sperm can shape the offspring phenotype is an issue that remains largely unsolved in cattle, owing to the delayed collection of performance data relative to gestation, calving, and lactation and to the confounding effect of genetics. The design of affordable epigenotyping tools that could be used on both semen and the blood of daughters, together with the development of integrated approaches that combine both genetic and epigenetic information, may help to address this question.

## About the Authors

Dr. **Hélène Kiefer** obtained a permanent position at INRA (now INRAE) as a research scientist in 2010. She joined a team working on livestock epigenetics and has since developed several tools to study DNA methylation at a genome-wide scale in cattle: a Roche-NimbleGen microarray and more recently RRBS. She collaborates with bioinformatics specialists and statisticians to develop pipelines for DNA methylation analyses. Her research aims to understand how epigenetic modifications vary under the influence of both environmental and genetic factors and contribute to phenotype variability in cattle. She is particularly interested in the bull sperm epigenome and its potential role as a mediator of intergenerational inheritance effects.

**Dr. Eli Sellem** has been working at Allice for 20 yr. He is particularly interested in the enhancement of semen production and semen quality, cryopreservation, and preservation and in predicting the onset of puberty. His key focus is on predicting bull fertility by developing sperm functionality assessments and in the past 7 yr through analysis of the semen epigenome.

**Dr. Amélie Bonnet-Garnier** is a senior scientist working at the French National Research Institute for Agriculture, Food and Environment (INRAE) in the Biology of Reproduction, Epigenetic, Environment and Development Unit (BREED, UMR 1198, INRAE UVSQ ENVA, UP Saclay). Her work focuses on deciphering how genome organization and epigenetic mechanisms regulate gene expression at the time of embryonic genome activation. She has developed technologies using FISH on 3D-preserved nuclei and image analysis approaches to study the links between large-scale chromatin reorganization and developmental success in several mammalian embryos.

**Dr. Maëlle Pannetier** obtained her PhD in molecular genetics at the University of Versailles Saint-Quentin, working on sex determination in the goat species at INRAE in Jouy-en-Josas. During her postdoctoral attachment at the Institute of Molecular Genetics in Montpellier (CNRS), she developed her skills and interest in the epigenome, and particularly the histone posttranslational modifications involved in parental imprinting control. She joined INRAE as a research scientist in 2008. She currently manages a small group in the BREED laboratory that works on sex determination and gonad differentiation in ruminant and rabbit species. They are focusing on the differentiation of germ cells and somatic cells from the physiological, genomic, epigenomic, and transcriptomic points of view.

**Valentin COSTES** is a PhD student from the cooperative union Allice and is working in the host laboratory BREED (Biology of Reproduction, Environment, Epigenetics and Development) at INRAE and Paris-Saclay University. His PhD project consists of the identification of sperm epigenetic biomarkers for different phenotypes in the bovine species, using integrative and modeling approaches.

**Dr. Laurent Schibler** is currently the head of Allice’s Development & Innovation team. After an agricultural engineering degree and a PhD in molecular genetics and structural genomics, he worked for 15 yr at INRA, at the crossroad of several disciplines but mainly on animal genetics and genomics; he thus contributed to the identification of several genes and QTLs of agronomic interest. As a team leader, he has used state-of-the-art genomics and proteomics technologies to study cartilage maturation in several transgenic mouse models and to offer new insights into the molecular mechanisms involved in equine osteochondrosis. His current aim is to develop integrated projects in cattle reproduction, focused in particular on semen fertility and combining genetics, (epi)genomics, and physiology.

**Dr. Hélène Jammes** is an expert in reproductive physiology. In 1984, she started working at INRAE on the role of gonadotropin in luteal cell activity and growth hormone (GH) transduction signaling in the mammary gland. In 2002, she developed epigenetic analyses of imprinted genes related to human pregnancy pathologies. She has worked on the epigenetic effects induced by Assisted Medical Procreation on embryo and placenta development using mouse models. She has investigated DNA methylation alterations in sperm from infertile men. A little over 10 yr ago, she returned to INRAE and joined UMR 1198 (Biology of Reproduction, Environment, Epigenetics and Development) and currently manages a team of eight permanent staff and students. This team focuses on epigenetic biomarkers for the programming of phenotypes. She also leads studies on DNA methylation as a biomarker for dairy cow health.
